# Taste function in children: normative values and associated factors

**DOI:** 10.1038/s41390-021-01920-w

**Published:** 2021-12-28

**Authors:** Mirjam van den Brink, Irene IJpma, Marta Fiocco, Wim J. E. Tissing, Remco C. Havermans

**Affiliations:** 1 Laboratory of Behavioural Gastronomy, Centre for Healthy Eating and Food Innovation, Maastricht University Campus Venlo, Venlo, the Netherlands; 2 Princess Máxima Center for Pediatric Oncology, Utrecht, the Netherlands; 3 Mathematical Institute, Leiden University, Leiden, the Netherlands; 4 Medical Statistics, Department of Biomedical Data Science, Leiden University Medical Center, Leiden, the Netherlands; 5 Department of Pediatric Oncology and Hematology, University of Groningen, Beatrix Children’s Hospital, University Medical Center Groningen, Groningen, the Netherlands

## Abstract

**Background:**

Although less frequent than in adults, taste loss also occurs in childhood. “Taste Strips” are frequently used for diagnosing taste dysfunction; however, normative values are lacking for children. In this study, we will create normative values for the “Taste Strips” in children.

**Methods:**

This cross-sectional study included 609 children aged 6–15 years. “Taste Strips” were used to determine sweet, sour, salty, and bitter taste scores by a non-forced procedure. The 10th percentile was used to distinguish normal taste function from a reduced sense of taste. Multivariable generalized linear models (GLM) were estimated to study the effect of age (group), sex, and 6‐n‐propylthiouracil (PROP) status on taste function.

**Results:**

Taste function changed with age, allowing for a distinction of three age groups: (I) 6–7 years, (II) 8–9 years, and (III) 10–15 years. Normative values were created for the age groups and boys and girls separately. Additionally, GLM showed a significant effect of (1) age (group) on sweet, salty, bitter, and total taste scores; (2) sex on sweet, sour, and total taste scores; and (3) PROP status on total taste scores.

**Conclusions:**

This study provided normative values for the “Taste Strips” in children, highlighting age- and sex-related differences.

**Impact:**

- Taste dysfunction can be harmful and impacts quality of life, a topic that became increasingly important since the COVID-19 pandemic.
- Although taste dysfunction is thought to be rare in childhood, the detrimental impact of such dysfunction might be large, as children’s eating habits are strongly influenced by input from the chemical senses.
- Measuring taste function may elucidate the relationship between taste dysfunction and disease, fostering the development of more appropriate supportive strategies. However, adequate tools are lacking for children.
- Normative values of the “Taste Strips” are now available for children, which bolster the clinical utility of this test.

## Introduction

Little is known about taste dysfunction in children. In general, it is thought to be rare as chemosensory problems are typically associated with aging.^1^ Nevertheless, children are not safeguarded from health problems that affect their smell and taste function. Medical conditions such as cancer, diabetes mellitus, kidney disease, and obesity are only a few examples of etiologies associated with chemosensory dysfunction in children, consequently affecting food choice, dietary intake, and overall health status.^2–6^ Measuring taste function may elucidate the exact relation between taste dysfunction and disease, fostering the development of more appropriate supportive strategies (e.g., medication, dietary advice) in treating disease.^7^ Therefore, it is not a matter of debate that clinical assessment of taste function in children is needed.

In contrast to smell tests, few taste tests are available in a clinical setting. In part, this is due to the low prevalence of taste disorders (relative to smell disorders).^8^ Indeed, most patients complaining about taste dysfunction actually suffer from smell dysfunction.^9^ The “Taste Strips” test is a frequently employed and well-validated clinical taste test.^10^ Advantages of this test are its long shelf-life, easy administration, commercial availability, and short time of investigation. In the context of research, the “Taste Strips” have already frequently been used in children.^11–13^ However, normative values are hitherto restricted to individuals aged 15–87 years.^10,14^ Especially within a clinical setting, if taste function is expected to be compromised as a result of disease burden and/or treatment, one needs to be able to determine a child’s taste function and interpret these scores by using population specific normative values.

Several factors can modulate taste function in children that should be taken into account and needs further investigation concerning the “Taste Strips.” First of all, taste function seems to improve as a child grows older, although results differ per tastant. For example, sucrose intensity has been reported to increase from childhood to adulthood, but sensitivity to bitter compounds was found to be similar between children and adults.^15,16^ Secondly, although most studies found no clear sex-related differences in taste function in children, there is some indication that girls outperform boys.^17–20^ Thirdly, the ability to taste bitter compounds such as 6-n-propylthiouracil (PROP), which can be partly explained by genetic variations in TAS2R38 polymorphisms, correlates with an increased intensity perception to taste stimuli and other orosensory sensations.^21,22^ However, it remains unclear whether PROP status is associated with taste scores as measured by “Taste Strips” in children.

Thus, the present study aimed to (1) provide normative values for the “Taste Strips” from a large sample of healthy children, (2) study the effect of age, sex, and PROP status on taste scores in children, and (3) identify to what extent scores on the “Taste Strips” test are associated with self-reported taste function in children.

## Methods

### Participants

This study was performed at the NEMO Science Museum Amsterdam, the Netherlands. All consecutive visitors to the museum were asked to participate. Participants were eligible for participation if they were between 5 and 17.99 years of age, able to understand Dutch or English, and reported to be healthy. Exclusion criteria for participation were: having a cold, smoking, being pregnant or a self-reported allergy to quinine. Parents provided written informed consent.

### Assessment of taste function

#### Taste Strips

Taste function was assessed by using “Taste Strips” (Burghart, Wedel, Germany). “Taste Strips” are filter-paper strips impregnated with a taste solution and determine sweet, sour, salty, and bitter taste scores. Four concentrations of each taste quality were used: sweet (0.05, 0.1, 0.2, and 0.4 g/ml sucrose), sour (0.05, 0.09, 0.165, and 0.3 g/ml citric acid), salty (0.016, 0.04, 0.1, and 0.25 g/ml sodium chloride), and bitter (0.0004, 0.0009, 0.0024, and 0.006 g/ml quinine hydrochloride). To each child, 16 impregnated strips and 2 blank strips were presented in the same pseudo-randomized order starting with the lowest concentration.

Children were asked not to eat and drink except water 1 h before the test. Before the test began, taste qualities were explained by presenting photographs (i.e., sweet like sugar, sour like lemon, salty like salt, bitter like coffee). “Taste Strips” were placed on the middle of the tongue, approximately 1.5 cm from the tip. Participants were then asked to close the mouth and indicate whether the perceived taste was sweet, sour, salty, bitter, or tasteless. Scores for each taste quality range from 0 to 4 and the total taste score was derived by summing the scores of each taste quality (range 0–16). A higher score represents a better taste function.

#### PROP test

PROP taster status was determined by a filter paper strip impregnated with PROP (Sensonics International, NJ, United States, 20 µg/strip). Participants were instructed to place the strip on the dorsal surface of the tongue for approximately 30 s and were then asked whether they tasted anything (yes/no).^23^ Participants who answered “no” or reported that the strip “tastes like paper” were classified as “non-tasters.” Children who indicated that the strip tasted “bitter,” “sour,” “bad,” or “spicy” were classified as “tasters.” In addition, participants who immediately removed the strip because of its “foul” taste or showed other signs of taste rejection were classified as “tasters” as well.^24^

### Self-report

Participants were asked to self-assess their taste function by rating their taste perception (on a 10-point scale: 1 “very bad” to 10 “very good”) and by estimating their taste function relative to their peers on a 5-point Likert scale (1 “much worse than others” to 5 “much better than others”).

### Statistical analysis

Descriptive statistics are presented as median with interquartile range (IQR) and the number of participants (n) with percentage (%). The 10th percentile was used to distinguish normal taste function from a reduced sense of taste (i.e., hypogeusia).^10^ Non-parametric tests were used to assess differences between sex, age or self-reported measures in relation to taste scores. Bonferroni post hoc procedure to adjust for multiple testing was used when required. To study the association between taste scores (i.e., sweet, sour, salty, bitter, and total) and the independent variables age (group), sex, and PROP status, multivariable generalized linear models (GLM) were estimated since the dependent variables were not normally distributed. To study the association between subgroups and outcome, interaction terms were created and included in the model if significant. An alpha level of 0.05 was used. Data analysis was performed with IBM SPSS Statistics (version 25.0).

## Results

### Participant characteristics

**Fig. 1:**
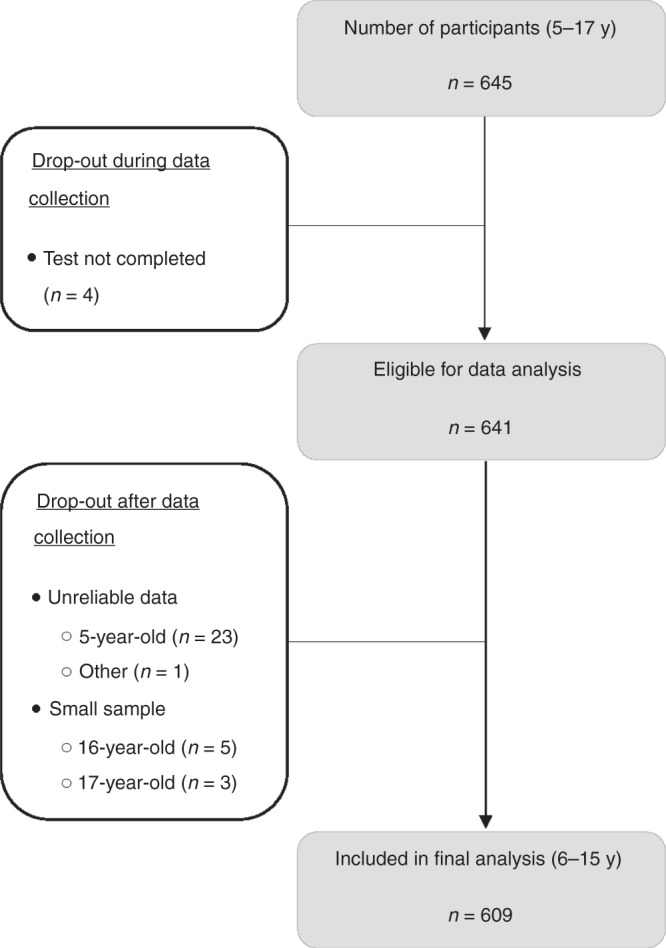
Flowchart of the included children in the current study.

In total, 645 children aged 5–17 years participated. Figure 1 shows a flowchart of the inclusion process. Four children did not complete the taste test. Among the 5-year-old children, identification rates for the highest concentration of sweet, sour, salty, and bitter taste were only 70%, 57%, 35%, and 61%, respectively, implying that the concentrations used in the “Taste Strips” test does not provide reliable results for <6-year-old children. Results of 5-year-old children were therefore excluded from analysis (*n* = 23), as well as the small number of 16-year-old (*n* = 5) and 17-year-old participants (*n* = 3). Thus, 609 participants aged 6–15 years were included for final analysis.

Participants’ mean age was 9.3 ± 2.3 years; 365 of the participants were girls (60%). Overall, 541 participants (89%) were living in the Netherlands, 56 (9%) were living in other European countries, and 12 (2%) came from non-European countries.

### Taste test results

Median scores (IQR) for the individual taste qualities were as follows: sweet 4.0 (3.0–4.0), sour 3.0 (2.0–3.0), salty 4.0 (2.5–4.0), and bitter 4.0 (3.0–4.0). Median total taste score (IQR) was 13.0 (11.0–14.0). In total, 373 children (61%) were PROP tasters.

**Fig. 2:**
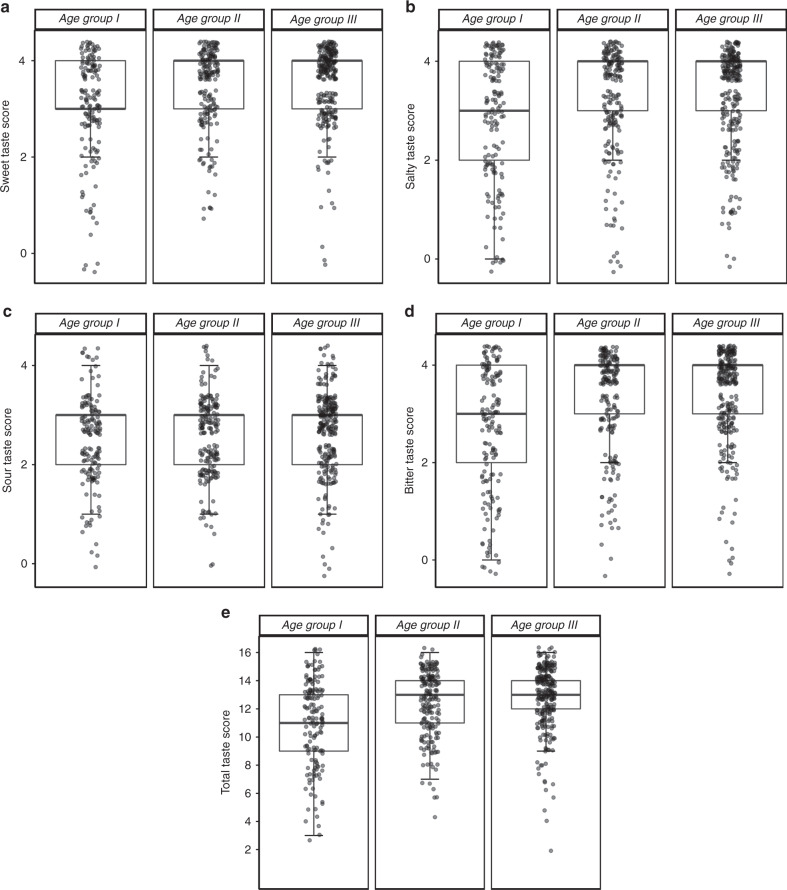
“Taste Strips” scores for the different age groups. **a** Sweet taste, **b** salty taste, **c** sour taste, **d** bitter taste, and **e** total taste score. Boxplots refer to the median score (midpoint of the scores), the first quartile of the scores (Q1, lower boundary of the box) and the third quartile of the scores (Q3, upper boundary of the box). The range of the box represents the interquartile range (IQR = Q3 − Q1) and the whiskers indicate what data points can be considered as outliers. The upper whisker extends to the most extreme score no more than 1.5 times the IQR above Q3, and the lower whisker extends to the most extreme score no more than 1.5 times the IQR below Q1. Note that, due to the limited range of possible scores for the individual taste qualities (0–4; **a**–**d**), some boxes (and whiskers) appear constricted.

A sex difference (*p* = 0.001) and positive correlation *(r* = 0.247, *p* < 0.001) between age and total taste score was observed, showing major age- and sex-related differences between 6 and 9 years of age. Based on these results, three arbitrary age groups were distinguished: group I, 6–7 years; group II, 8–9 years; group III, 10–15 years. A significant difference in sweet (*p* < 0.001), sour (*p* = 0.044), salty (*p* < 0.001), bitter (*p* < 0.001), and total taste score (*p* < 0.001) was found between the three age groups (Fig. 2). Post hoc testing indicated significant differences between group I and II and group I and III for sweet, salty, bitter, and total taste scores (*p* < 0.001). For sour taste, no significant differences were found between age groups when adjusted for multiple testing.

### Normative values

Table 1 shows the distribution of taste scores separated for age and sex. According to the 10th percentile, a total taste score below the following values indicate hypogeusia: group I, <7.0 for girls and <6.0 for boys; group II, <10.0 for girls and <8.0 for boys; and group III, <9.9 for girls, and <10.0 for boys.

**Table 1:** Distribution of taste scores separated for age and sex (*n* = 609).

		Sweet score		Sour score		Salty score		Bitter score		Total score	
		Boys	Girls	Boys	Girls	Boys	Girls	Boys	Girls	Boys	Girls
Age 6–7 years											
*N*		65	89	65	89	65	89	65	89	65	89
Minimum		0	0	0	0	0	0	0	0	3	3
Maximum		4	4	4	4	4	4	4	4	16	16
Percentile	10	1.0	2.0	1.0	2.0	1.0	1.0	1.0	0.0	6.0	7.0
	25	2.0	3.0	2.0	2.0	2.0	2.0	1.5	2.0	8.0	9.0
	50	3.0	3.0	2.0	3.0	3.0	3.0	3.0	3.0	11.0	12.0
	75	4.0	4.0	3.0	3.0	4.0	4.0	4.0	4.0	13.0	13.0
	90	4.0	4.0	3.0	4.0	4.0	4.0	4.0	4.0	14.0	15.0
Age 8–9 years											
*N*		65	128	65	128	65	128	65	128	65	128
Minimum		1	1	0	1	0	0	0	0	4	6
Maximum		4	4	4	4	4	4	4	4	16	16
Percentile	10	1.6	3.0	1.0	2.0	1.0	2.0	1.0	2.0	8.0	10.0
	25	2.0	3.0	2.0	2.0	2.0	3.0	2.0	3.0	9.5	11.3
	50	3.0	4.0	2.0	3.0	3.0	4.0	3.0	4.0	12.0	13.0
	75	4.0	4.0	3.0	3.0	4.0	4.0	4.0	4.0	14.0	14.0
	90	4.0	4.0	3.0	4.0	4.0	4.0	4.0	4.0	15.0	15.0
Age 10–15 years											
*N*		114	148	114	148	114	148	114	148	114	148
Minimum		0	0	0	0	0	0	0	0	2	4
Maximum		4	4	4	4	4	4	4	4	16	16
Percentile	10	3.0	3.0	2.0	2.0	2.0	2.0	2.0	2.0	10.0	9.9
	25	3.0	3.0	2.0	2.0	2.8	3.0	3.0	3.0	12.0	12.0
	50	4.0	4.0	3.0	3.0	4.0	4.0	4.0	4.0	13.0	13.5
	75	4.0	4.0	3.0	3.0	4.0	4.0	4.0	4.0	14.0	15.0
	90	4.0	4.0	4.0	4.0	4.0	4.0	4.0	4.0	15.0	15.0

### Effect of age, sex, and PROP status on taste scores

Results based on the multivariable GLM models show the effect of age, sex, and PROP status on sweet, sour, salty, bitter, and total taste scores (Table 2). Adjusted for sex and PROP status, total taste scores of children aged 8–9 years and 10–15 years were, respectively, 1.3 and 1.8 points as high as children aged 6–7 years. In addition, higher salty and bitter scores were found in children aged 8–9 years compared to 6–7-year-old children and higher sweet, salty, and bitter scores were found in children aged 10–15 years compared to 6–7-year-old children when adjusted for sex and PROP status.

**Table 2:** Estimated parameters and confidence intervals based on GLM for dependent variables sweet, sour, salty, bitter, and total taste scores.

	Sweet score (0–4)	Sour score (0–4)	Salty score (0–4)	Bitter score (0–4)	Total taste score (0–16)
*Independent variables*					
Age (reference: 6–7 years)					
8–9 years	0.21 (−0.08; 0.49)	−0.01 (−0.20; 0.16)	0.30 (0.05; 0.54)*	0.38 (0.15; 0.61)*	1.33 (0.75; 1.91)*
10–15 years	0.64 (0.38; 0.90)*	0.16 (−0.01; 0.33)	0.34 (0.11; 0.57)*	0.52 (0.30; 0.74)*	1.83 (1.28; 2.38)*
Sex (reference: boys)					
Girls	0.40 (0.13; 0.67)*	0.20 (0.02; 0.30)*	0.08 (−0.11; 0.27)	0.15 (−0.03; 0.33)	0.71 (0.25; 1.17)*
PROP status (reference: non-tasters)					
Tasters	0.07 (−0.08; 0.22)	0.06 (−0.08; 0.20)	0.08 (−0.11; 0.27)	0.14 (−0.04; 0;32)	0.62 (0.16; 1.01)*

Note: for sweet taste, the interaction between age and sex was significant and included in the model, showing an opposite effect for girls aged 10–15 years (*B* = −0.40, −0.80; −0.03).

*p < 0.05.

Girls outperformed boys by showing a total taste score of 0.7 points as high as boys but also exhibit higher sweet and sour scores, when adjusted for age and PROP status. Except for sweet taste, the interaction between age and sex on the outcomes was not significant, implying that there is no different effect among subgroups on sour, salty, bitter, and total taste scores.

PROP status was associated with total taste score, with “tasters” having a total taste score of 0.6 points as high as “non-tasters,” when adjusted for age and sex. Neither PROP status and age nor PROP status and sex showed a significant interaction with the outcomes.

### Self-report

The mean rating for taste function was 7.7 ± 1.4. No significant correlation was found between this rating and total taste scores in children. Additionally, self-reported taste function was rated to be “much better than others” by 18 participants (3%), “better than others” by 125 participants (21%), “similar to others” by 447 participants (73%), “worse than others” by 16 participants (3%), and “much worse than others” by 3 participants (<1%).

## Discussion

This is the first study that provides normative data for the “Taste Strips” from a large sample of children, divided into three age groups (6–7, 8–9, and 10–15 years) and separated for sex. The present study also shows that taste scores (1) increased with age for sweet, salty, bitter, and total taste; (2) was higher in girls compared to boys for sweet, sour, and total taste scores; (3) was higher for PROP “tasters” compared to “non-tasters” regarding total taste scores; and (4) did not correlate with self-report.

Age and sex differences in taste function among children were also found by James and colleagues, who showed higher detection thresholds for sucrose and sodium chloride in 8–9-year-old boys compared to 8–9-year-old girls when using freshly prepared taste solutions.^20^ In addition, they compared 8–9-year-old children’s taste function with adults, showing no significant differences in taste function between 8–9-year-old girls and adults, but 8–9-year-old boys showed a poorer ability to detect sweet, sour, and salty taste than adult males and a poorer ability to detect all four taste qualities than adult females. A similar trend can be found in the current study, when comparing children’s taste scores with those previously found among adults.^10^ Our results corroborates prior findings, suggesting that taste is functionally mature around an age of 10 years and that taste function matures a bit faster in girls.^20,25^

PROP status was associated with taste function in children, with higher total taste scores in “tasters’ relative to “non-tasters.” Although significant, this difference was moderate (0.6 points) and was not found for individual taste qualities. This is in line with a study that studied five common ways of measuring taste function (i.e., detection thresholds, recognition thresholds, suprathreshold intensity, PROP bitterness, and fungiform papillae) in women.^26^ They found that detection thresholds and recognition thresholds for sweet, sour, salty, bitter, and umami did not correlate with PROP bitterness, but also not with fungiform papillae density. In general, only detection and recognition thresholds were related, which highlights the complexity of identifying taste function. Thus, the current study found PROP status to be associated with taste function, but not to the extent that it can be seen as a measure of overall taste function in children.

Self-reported taste function was not correlated with total taste scores. This indicates that children are not aware of their own taste function (or indeed dysfunction). One might argue that the absence of a correlation between self-reported taste function and performance on the “Taste Strips” test indicates that the “Taste Strips” are simply not valid, at least not in children. However, this also appears to be the case in adults as self-report of taste dysfunction has been shown inaccurate when using focused questions.^27^ For that reason, self-reported taste abilities, which can provide meaningful information to the clinician, should be always accompanied by a more objective test such as the “Taste Strips.”

Two methodological choices should be noted. First of all, a non-forced choice paradigm was used, according to the original protocol for the “Taste Strips.”^10^ This allows the participant to indicate a strip as “tasteless,” instead of being forced to choose between sweet, salty, sour, or bitter. Forced choice testing has the advantage of limiting response bias and malingering.^8,28,29^ The major disadvantage of a forced-choice method, however, is the inability to determine whether a “hit” or “miss” reflects an individual’s taste function or random guessing. As this information might be useful for the clinician if taste sensitivity is expected to be lost or changed, a non-forced choice method was chosen. Secondly, the 10th percentile was used to separate normogeusia from hypogeusia, according to the original protocol and originating from research into adults’ smell function.^10,14,29,30^ As we do not know how many children suffer from taste dysfunction, though presumably <10%, using the 10th percentile could lead to false-positive results and overdiagnosis. Using two standard deviations from the mean might be an alternative and more conservative approach.^31^ However, if this remains unclear, we have chosen to apply the commonly used 10th percentile as a cut-off value for children’s taste function, additionally considering their self-report and clinical symptoms.

Strengths of our study were the large sample size and wide age-range of included participants. In addition to Dutch children, also children from other European countries were included which increases the generalizability of our findings. However, some limitations should be noted. As mainly European children were included, it is uncertain whether these normative values extend to children outside of Europe. Furthermore, body weight or body mass index was not reported or measured, which could be a confounding factor as obesity is associated with a lower taste sensitivity in children.^11^

In conclusion, normative values for the “Taste Strips” were obtained for children aged 6–15 years, highlighting age and sex differences. These results bolster the clinical utility of the “Taste Strips” among children in a clinical setting, beyond its easy and quick administration.
